# Practical Medicine and Therapeutics

**Published:** 1837-01

**Authors:** 


### PRACTICAL MEDICINE AND THERAPEUTICS.

*On Debility in Nervous Diseases, and particularly on the Employment of Tonics in Insanity.* By DR. GUISLAIN, chief Physician of the Institution for the Insane at Gand.

IT is well known that many symptoms, as pain, convulsions, disordered intellect, &c., may be developed under the influence of debilitating as well as of exciting causes. Thus, nausea and vomiting may be produced both by inflammation of the stomach, and by the constant use of watery and mucilaginous drinks. Prolonged abstinence will bring on acute pains in the stomach, which are symptoms also of inflammation, and ulceration of that organ. Anxiety, palpitation of the heart, cough, and dyspnœa, may be the effect of undue lactation, abstinence, low diet, &c. as well as symptoms of pulmonary inflammation. Hard drinking will cause mania, as well as abstinence from drink in confirmed drunkards. Want of sleep, convulsions and tinnitus aurium, are symptoms of cerebral irritation, and may be brought on by great loss of blood. Frontal headach may depend on inflammation of the meninges, indigestion, repletion; but it may follow great losses of blood, or prolonged fasting.

Among the same class of facts may be included those cases in which medicines differ in their action, although given under circumstances apparently identical. Thus, cardialgia attended with intolerable suffering is aggravated by antiphlogistics, and relieved by sedatives and tonics. Aromatics, volatile oils, ethers, relieve nervous enteralgias, and the greater number of convulsive diseases yield, not to debilitants, but to tonics, and diffusible stimuli. Irritation, produced by one cause, may affect at the same time the nervous and the vascular systems, and may require, on the one hand, remedies to soothe the pain or to arrest the periodical returns of the disease; and, on the other, means calculated to remove the inflammatory condition. This mixed treatment, consisting of the antiphlogistic at first, and the anti-nervous subsequently, is required in a multitude of cases; as in many cerebral diseases, in affections of the nervous structure in those whose temperament depends on a preponderance of the nervous system, in intermittent fevers, and in acute rheumatism, which so often resists antiphlogistics and yields to narcotics and bark. In the treatment of all mental diseases, it is important to recognize the period at which they pass from an active to a passive state; at this latter period the disease only consists in nervous mobility, which is kept up by the functional habits of the nervous system.

M. Barras has observed that a correct idea cannot be formed of the neuroses, if we confine our observation to their external characters only, as a disease may put on the form of great excitement, although it depend on a state of debility. In treating the insane, the same phenomena which indicate the employment of febrifuge tonics in intermittents may guide us; and these may be referred to a peculiar state of the sanguineous system, marked by paleness of the lips, borders of the eye-lids and nostrils; there is also some emaciation, pale urine, pulse frequent but soft, expression anxious, conjunctiva blueish, even sky-blue. This state of the eye is observed in all feeble and delicate men; it belongs to the nervous temperament; it is marked in women with leucorrhœa, or exhausted by lactation, or hemorrhage, and in the insane who have been exposed to debilitating agents. Thus Dr. G. has observed it constantly in those who refuse to eat.

The frequency of the pulse among the insane is in a direct ratio to the mental disturbance; in mania it is less rapid where there are symptoms of cerebral congestion. In the asthenic period of the disease, its frequency is extreme, and counted with difficulty. After quinine, alone or combined with opium, has been given, the pulse increases in size and diminishes in frequency, and in the same proportion the cerebral disturbance is quieted, the loquacity decreases, sleep returns, and convalescence, when the pulse has regained its healthy type. Sometimes in mania the pulse is extremely slow, and in these cases quinine is less serviceable than where there is great frequency.

The indication for tonics in insanity is not to be exclusively formed from the disease having been preceded by debilitating causes, such as prolonged lactation, bleeding, &c., although they powerfully increase cerebral irritability; for weakness of the nervous system is also independent of these causes, and is most frequently the result of increased action. This active stage, however, cannot be considered as one of pure inflammatory irritation, as the treatment proves. A debilitating regimen and emollients calm pain in the active period of neuroses, and sometimes remove them; but, in the majority of cases, bleedings, leeches, and antiphlogistics only increase morbid irritability, and particularly at an advanced period of the disease; whilst experience proves the benefit of tonics. It is astonishing, as this is the case, that they should not have been more used in mental diseases.

As practitioners have generally withheld tonics from the insane, from a fear of a congestive or inflammatory condition of the brain, Dr. Guislain attempts to distinguish between insanity with congestion and with mere morbid nervous mobility. This latter condition is characterized by an exaggeration of the functions of the brain. There is no moderation in the manifestation of the feelings, passions, will, ideas of such patients; a trifle awakens their suspicion and hatred; they weep and laugh almost at the same time; the mobility of their features is excessive; they are very talkative, their muscular force is prodigious, and all their acts of locomotion, although prompt and violent, are admirably regulated: their memory is acute, and their intellects seem momentarily clear and elevated.

In the congestive condition, the acts of the brain are weakened in proportion to the superabundance of blood in the organ. This fluid oppresses the nervous functions, and at the same time becomes a cause of irritation. This is the case in all inflammations. When the retina is congested, the sight is impaired; whilst, in some nervous affections of the membrane, its functions become more acute. In coryza, the smell is lost, but there is an increased sensibility to odours in many nervous affections attended by debility. In gastritis, there is no appetite, as digestion is impossible; whilst, in the most painful cardialgia, the appetite remains. In the congested state of the brain in insanity, there is a gradual extinction of sensibility: the patient expresses no pain when irritated; he fixes his eyes without seeing, and listens without hearing; he has neither fear nor aversion; neither will nor physical force; his features are immoveable; his utterance slow, confused, or impossible; his intellectual powers are equally weakened; there is a progressive extinction of his feelings, memory, and ideas, with inclination to comatose sleep.

Between these two extremes there are mixed states, the patient being at the same time in a state of maniacal excitement, whilst his memory is failing, his walk vacillating, and his muscles slightly convulsed. In all cases the state of the tongue throws light on the diagnosis: whenever there is cerebral congestion, its motions are embarrassed; hence the importance of attending to the pronunciation when there is a question as to the propriety of tonics. In those cases which yield to tonics, the amelioration is gradual, the cure taking place some weeks, or even months, after the remedy has been persisted in even in large doses.

Case 1. Amelie T., æt. thirty-six, entered the Institution the 27th of March,

1834, in a state of violent mania, characterized by cries, vociferations, and incoherent actions. She was a widow with seven children, the youngest of whom was illegitimate, and only seventeen days old. During her confinement, and for some time previously, she had been subjected to great privations, and she had been much alarmed during parturition: the intellectual disturbance commenced suddenly seventeen days afterwards. From the 27th of March to the 2d of April, she was placed in seclusion, and the mania was marked by complete incoherence of speech, hoarseness, cries, fury, weakness of the pulse, dirty complexion, blue colour of the conjunctiva, and dilatation of the pupils. Dr. Guislain prescribed solitude and a tonic regimen; meat jellies, and twenty grains of sulphate of quinine daily. After eight days of this treatment, there was a sensible improvement; the expression of the eyes became natural, the convulsive state of the features gave way to an expression of good humour; conversation rational, voice weak, pulse frequent.—April 15th. The amelioration is progressive, she sleeps well, and quietly; pupils contracted, cheeks flushed, pulse slow; she complains of her head feeling empty. Owing to a mistake, a relative was admitted, who reproached her, and she became agitated, could not sleep, and fell into a state of melancholy. By great care, substantial food, and decoction of bark she was restored, and quitted the establishment on the 20th of July in perfect health.

Case 2. A man, æt. sixty, had been an in-patient in an hospital for upwards of a year for an asthmatic affection, when, in June, 1834, he became maniacal, and was placed under the care of Dr. G. Day and night he disturbed the other patients by his loquacity, his cries, and songs; his ideas were completely incoherent. Pulse frequent, small, tremulous; urine clear, features contracted, eyes prominent, sparkling, conjunctiva blue, temperature of the skin diminished. He was ordered to take two drachms of extract of bark daily. This was so successful, that, on the third day, the disturbance of his intellect was removed. The medicine was continued during two months; but, as soon as the cerebral symptoms ceased, the chest became again affected, and he died eight months afterwards, without having experienced any return of mania.

Case 3. N. D., eighteen years of age, had an attack of mania, which lasted six months. At twenty-two years, the political disturbances again brought it on, and this time it put on an intermittent form, returning every eighteen days, and lasting about a fortnight. The attack began with sadness for a day or two, followed by childish gaiety and constant laughter; and this state increased until his fantastic impulses led him to destroy his clothes and break the furniture. On the tenth or eleventh day, he became drowsy, which state terminated in health. During the lucid intervals, his intellect was perfect, and he was able to attend to business, and to enjoy public amusements. The return of the disease was so regular, that these intervals were never prolonged beyond eighteen days. The disease had lasted four years when Dr. G. was consulted. Bleedings, leeches to the head and arms had been frequently and copiously resorted to, without the least success. He directed two scruples of sulphate of quinine to be taken during each interval, and this was persisted in for four successive months. One grain of extr. opii was taken daily during the same period. This at first diminished the intensity of the attacks without arresting them, but suddenly, in September, 1834, they ceased, and he is now perfectly well.

Several other successful cases are detailed; but Dr. G. observes that he does not recommend quinine as a certain panacea in all instances, as he has employed it many times without benefit. Sometimes there was a tendency towards convalescence, which the bark hastened. In no case did it aggravate the cerebral symptoms, although the doses were very large and long continued. It is astonishing that furious maniacs can take beer, wine, or even brandy, without aggravating their disease; indeed, in some cases there is relief after slight intoxication, and the effect of wine and opium in the mania of drunkards is well known. On the other hand, all those who are practically acquainted with insanity have seen cases aggravated by bleeding; and, notwithstanding this, it is often resorted to with blind confidence. It may be useful to prevent cerebral congestions and disorganization, and at the commencement it has arrested the disease; but such cases are rare, and, during fifteen years' particular attention to insanity, Dr. G. has not had ten cases of this sort. In the great majority of those who were bled more or less frequently, the symptoms have been aggravated; and, even although bleeding may apparently calm the cerebral irritability, yet generally it renders the disease incurable: even where there is cerebral congestion, it may arrest the tendency to coma, render the step more free, and remove muscular contractions; but does not affect the mental condition, and the intellectual disturbance increases. Dr. G. does not recollect to have cured one patient whose pronunciation was embarrassed, or walk vacillating. *Gazette Med. de Paris*, No. 14, *Avril*, 1836.

---

*On the diagnostic Value of the Deformities of the Chest, produced by Diseases of the Thoracic Organs. By* M. Woillez. (*Thesis.*)

The examination and measurement of the thorax of 116 men, in the wards, of M. Louis, have furnished M. Woillez with the materials for this memoir. The following are his principal conclusions:—Frequently the natural projections were similar in form to those resulting from disease. Natural projections are very rare on the front of the right side or on the back of the left side, but they are frequent on the back of the right side and front of the left side; these last two are often found together. In the healthy state, the right side measures more than the left: so that measurement of the chest is of little use in the diagnosis of dilatation of the thorax, unless the patient has been previously measured.

M. Woillez first demonstrates that, as the lung tends incessantly to contract by virtue of its elasticity, the dilatation of the chest can only be produced when this elasticity (or, as he calls it, this concentric force, by which the ribs are kept in contact with the lung,) is destroyed. In pneumonia, although the lung is evidently increased in volume, there is no enlargement of the thorax. This enlargement exists if the hypertrophied lung is sufficiently large, to project from the chest on opening the body. Projection of the chest takes place in almost all cases of emphysema: from what has been said above, this projection is more valuable as a sign on the right side in front. The disappearance of the intercostal space being always pathological, it is a good mark to distinguish these cases from natural projections. In pleurisy, it has been observed by all that the effused liquid does not accumulate inferiorly in the first stage; this our author explains by saying that the concentric force not being destroyed, there is a tendency to a vacuum in every point of the thorax. In the second stage, on the contrary, the elasticity of the lung is destroyed, the fluid accumulates in the lowest part, and, when it is in sufficient quantity, the chest is dilated. M. Woillez next examines the contractions which supervene after the cure of pleurisies, and explains very well the apparent irregularities which are met with. If, for example, the left side does not appear depressed after the cure of pleurisy, with considerable effusion on that side, it is owing to the existence of one of those frequent projections which the contraction has only diminished. The depression of the nipple is a good sign of an old pleurisy; for, in healthy individuals, in whom the nipples are not on the same level, the nipple which is most elevated is generally on the contracted side. Temporary projection of the thorax is a good sign of partial costo-pulmonary pleurisy. In effusions of air into the pleura there will neither be dilatation of the thorax nor compression of the lung, when the exit of the air is as free as its entrance. On the contrary, dilatation of the thorax takes place when the entrance of air into the pleura is easy and its escape difficult, or impossible. Little projections, having a dull sound, which precede aneurismal tumours of the large vessels, are valuable signs. The vaulted elevation, which is of little importance in recognizing hypertrophy of the heart, because it only takes place when the heart is enormous, is very useful in the diagnosis of pericarditis: of thirty-two cases of this disease, observed during four years by M. Louis, this elevation was noticed thirty-one times. M. Woillez says, it cannot be confounded with natural projections, as these never change, whilst those from pericarditis increase and diminish with the effusion. *Archives Gen. de Med. Juillet*, 1836.

*History of a Deaf and Dumb Person; with the Appearances on Dissection.* By Dr. BERGMANN.

A MAN was found hidden in a barn at Reinhausen, in the kingdom of Hanover, in the year 1806. All attempts to make him give, by word or sign, any account of himself, of his former life, abode, or circumstances, were vain. When interrogated, he laughed unmeaningly, or gazed about him fixedly, or uttered unintelligible sounds. He was of the middle size, thin, well made, and about twenty years old. All enquiries respecting him throughout the country having been without result, he was consigned to the workhouse at Celle. Here he lived till his decease, in 1833.

Soon after his arrival at Celle, he manifested signs of intelligence sufficient to prove that neglect and want of education had greatly contributed to render his case intractable. He was mild, good-natured, attentive and docile, obeyed at a sign, was extremely willing, and not unskilful in domestic service. He appeared cheerful and contented, and was very seldom out of temper. He had totally lost the faculty of hearing, and could only utter one or two sounds. However, he contrived to make himself tolerably well understood by gesticulation. Although, during the latter years of his life, his general appearance was healthy, he was still unusually weak, and suffered from shortness of breath. Dr. Bergmann has remarked that the deaf and dumb are peculiarly subject to weakness of the pulmonary organs; which weakness he easily deduces, *à priori*, from the relation of the latter to the organ of hearing, and from the intimate nervous connexion.

The patient died of a fever, with pulmonary symptoms, June 6, 1833.

The abdominal viscera were found, at the post-mortem examination, to be free from disease. In the thoracic there were pathological changes, corresponding to the symptoms manifested during the last years of the patient's life. The brain was in a normal condition, and its different parts exhibited no deviation from their usual structure. The external ear was of a natural conformation, and the meatus externus and membrana tympani presented no unusual appearances; but the membrane which lined the hollow of the tympanum was of a spongy, sarcomatous nature, and that which covered the bones of the internal ear was similarly affected, so that their motion might have been somewhat impeded in consequence. In the hollow of the tympanum of both sides there was a quantity of thickish mucus. A hard concretion, about the size of a mustard-seed, covered the fenestra rotunda on the left side. The semicircular canals were of a natural form, but degenerated internally; they seemed to have contained some fluid, but in very small and insufficient quantity. The thin, transparent lining membranes, (ductus semicirculares Scarpæ,) the ampullæ, and the nerves, could not be distinctly discovered on either side. The defects here were evidently of a nature to have interfered materially with the exercise of the function of the organ. They could not have been affected by the process of examination, for the appearances presented were precisely similar on both sides. The cochlea was found in its usual condition, and appeared to be properly supplied with nerves. Whether the defects visible in the semicircular canals were congenital or the result of disease, it was of course impossible to ascertain.

Dr. Bergmann contrasts with this case one of decided idiotism; that of a dumb girl, whose forehead was flat, head compressed on both sides, face without any expression, mouth constantly open, so that the saliva was always flowing out, look unmeaning, &c. In this case the brain was imperfectly developed, but all the organs of sense were of a natural conformation.

*Hannoversche Annalen für die gesammte Heilkunde*, B. i. Heft 1. 1836.

---

*Singular Case of Inflammation of the Muscles.* By J. A. PETET. (*Thesis.*)

CASE. A mechanic, about forty years of age, and of nervous temperament, accustomed to work hard, in a damp workshop, at his trade, which required much strength, began to suffer from general lassitude. This forced him to relinquish his occupation, and take to his bed; and shortly afterwards he experienced pains particularly in his limbs, exasperated by the slightest motion. The general symptoms were trifling; there was but little fever, moderate thirst, and no increase of heat; but he suffered greatly from want of sleep. On admission into the Hospital St. Louis, under the care of M. Biett, there was observed elongated, fusiform swellings in the direction of the length of the limbs, without change of colour or heat of the skin, but sensible on pressure, and particularly so on motion. The form of the tumours, which corresponded to that of the muscles, whose increased size caused them to be strangulated by their aponeurotic sheaths; the pains, which became unbearable on the least motion; and the absence of any alteration in the surrounding tissues, left no doubt but that the muscles were inflamed. The antiphlogistic treatment, together with some opiates, were the means employed; but the pains continued, and exhausted the patient, who died the second day after his admission, and about five days after the commencement of the disease. On examining the body, the sheaths of the muscles were found greatly distended, and an incision gave issue to a greyish, homogeneous pulp, in which it was impossible to distinguish the muscular fibres, or cellular tissue, vessels, or nerves. In some of these tumours, which were less advanced, the muscular fibres could be recognized, but they were evidently softened, and had lost their colour.

*Archives gén. de Méd., Juillet,* 1836.

---

*On Hypertrophy, with Dilatation of the Heart, in Children.*
By Dr. Toel, of Aurich.

Dr. Toel is of opinion that this affection frequently developes itself as a consequence of pulmonary inflammation, measles, hooping-cough, &c. It comes on insidiously, after the patient appears to have, in a great measure, recovered from one of the above complaints. It is a more frequent result if the latter have run an irregular course. Then, after some time has elapsed, the pulse, which had lost its inflammatory character, becomes quick, small, and sometimes irregular. A dry cough, without pain or expectoration, makes its appearance. The sounds of the heart are heard over a greater extent than usual; the strokes are not distinct, and would seem to flow into one another. The patient feels a weight and dull pain under the sternum, on the left side, but does not complain much of it. The breathing is shorter than formerly. The face assumes a very peculiar and almost indescribable expression of suffering. Towards evening the hands are perceptibly hotter; the jugular veins beat without intermission. The little patients are not yet thought to be very much out of health; they walk about, go to school, are only rather more delicate than usual, avoid an horizontal posture in bed, are very short of breath after exertion, and become more irritable than formerly. It is here, as usual, very difficult to define the point at which functional becomes structural derangement; but the latter may be confidently surmised when the symptoms continue without intermission, even during a period of perfect repose.

Dr. Toel recommends vigorous measures as soon as any of these symptoms make their appearance. The expectant method would lead inevitably to a fatal result. Antiphlogistic remedies must be adopted in full force, perfect repose both of mind and body enjoined, together with the absence of all stimulating, and the diminished exhibition of all nourishing agents. Should this stage of the disease be not combated vigorously and successfully, there is no hope for the future. The progress of the evil may be now slower, and now more rapid, and sometimes even be apparently arrested, but it is sure never to cease altogether. The symptoms which attend its advanced stage are too well known to need a new description. Dr. Toel's principal object is, to direct the attention of medical men to the fact that hypertrophy of the heart frequently follows the diseases above mentioned, as he is of opinion that such a result has not been sufficiently known, and consequently not sufficiently guarded against.

[A good deal of experience leads us to consider the preceding observations as of much practical importance. We believe that, in the majority of cases referred to by Dr. Toel, the primary affection of the heart is inflammation of the lining membrane of the cavities.—*Endocarditis.*]

*Hannoversche Annalen,* B. i. Heft 2. 1836.

*On the Therapeutic Value of Extracts of the Solaneæ containing Green Fecula.*
By MM. MARTIN-SOLON and SOUBERAIN.

EXTRACTS prepared from juices which have not been purified, and containing consequently the green fecula of the plants, have been recommended by Störck, and generally considered as useful medicines. In the greater number of plants, what is improperly called green fecula consists of a mixture of chlorophyle, of coagulated gluten, and of the debris of tissue,—substances of no medicinal value; but it would be wrong to conclude on this account that the green fecula of *all* plants is useless: it, however, may be doubted whether the opinion of those pharmacologists is correct who consider this fecula as the only active part of this family of plants, and do not hesitate to substitute it for the extract itself. MM. M. and S. have made experiments on the properties of the green fecula of belladonna, hyoscyamus, and stramonium, in order to determine whether it is a necessary ingredient in the common extracts, or if it could be separated without injuring their virtues. The experiments were made with two kinds of green fecula; the first, which may be called the insoluble matter of the juices, is that which is held in suspension after the tissue of the plant has been broken down, and been submitted to expression. It was collected by filtering the juice, and purified by several washings. The second kind, which is called green fecula, obtained by coagulation, is procured from the filtered juice after it has been heated. It contains, in addition to the ingredients composing the first species, albumen which is coagulated by the heat, and some matters entangled by the albumen during coagulation. In order to divide the doses equally, the fecula was, when still moist, mixed with sugar, and then dried in a stove.

*Insoluble Green Matter of Belladonna.* This was given to two patients: the first was a woman of sixty, with catarrh and chronic gastritis; one grain was given the first day, and the dose increased one grain daily. On the tenth day, the patient complained of muscæ volitantes: the dose was increased to fourteen grains without its producing any peculiar symptom whatever. The second patient was a woman, æt. thirty-two, convalescent from articular rheumatism: the dose was gradually increased to twenty grains, and no effect produced.

*Green Fecula of Belladonna obtained by Coagulation.* The first patient was a woman, æt. twenty, affected with rheumatic pains. Two grains were given at first, and the dose increased two grains daily. At fourteen grains, she complained of pain in the throat; she slept well, but dreamed more than usual, and had occasional spasms of the legs. When the dose was sixteen grains, she suffered much from her throat; her dreams were sad and frightful; her head heavy, pupils dilated, pulse regular; no rheumatic pains. The medicine was discontinued, and on the following day these symptoms entirely disappeared. The second patient was a phthisical young man of twenty: on taking twenty grains, he complained that his sleep was less tranquil than usual; no other symptom. In a third patient, æt. forty-four, phthisical, pain in the throat was complained of after taking ten grains; after fourteen, the pain increased, with slight headach; after sixteen, there was slight trembling of the hands, but the sleep was quiet. This tremor increased on augmenting the dose, and the medicine was discontinued.

*Insoluble Green Fecula of Hyoscyamus niger.* One trial was made in a young man, æt. twenty-two, convalescent from pneumonia. After taking twelve grains, he found some weakness of vision; his sleep was a little agitated.

*Green Fecula of Hyoscyamus obtained by Coagulation.* The patient was affected with habitual headach: dose augmented daily two grains. After taking ten grains, his sleep was disturbed; after fourteen grains, he slept more, but he complained of uneasiness in his legs; eighteen grains produced nausea, heaviness of the head, sleep disturbed by dreams. The medicine was omitted, and the day afterwards these symptoms ceased.

*Green Fecula of Stramonium.* Both kinds were given to the patients, and the dose carried to twenty grains, without any effect.

From these experiments, it may be doubted whether the green fecula which is left in the extracts of the plants with the juice can add to their efficacy: indeed, it is a question whether their activity may not be impaired by the mixture of the active principle with so much inert matter. It is not unreasonable to conclude, that the slight effects in some of the experiments were owing to a part of the soluble principles of the juices remaining mixed with the green fecula, particularly as the coagulated fecula was the most active, which was washed with less effect. These experiments, however, are not sufficient to decide the question. Störck prepared his extracts with a heat so gentle as not to injure the juices, instead of evaporating them, as was the general custom, by prolonged ebullition, to the great injury of their active ingredients. This may account for the success he and others experienced. *Bulletin général de Thérapeutique, Fevrier*, 1836.

---

*Tic Douleureux cured by the external Application of Tartrate of Antimony.*
By Dr. HAUSBRANDT.

A WOMAN, more than sixty years of age, had suffered for many years from face-ache, the severity and long continuance of which almost reduced her to despair. As soon as the pain of the face ceased, the patient felt comparatively well: when the pain came on, which was always suddenly and without any ostensible cause, the muscles of the face twitched, and the eye of the affected side was closed; the whole face became remarkably pale, and the features indicated severe suffering. As no particular circumstance capable of inducing the attack, excepting perhaps taking cold, could be discovered, the treatment was altogether empirical. A considerable number of remedies, such as are usually employed for this complaint, were tried,—especially frictions, vesicatories, narcotics, carbonate of iron,—but the paroxysms returned with greater frequency, and the patient not only lost flesh, but her condition seemed desperate. Dr. H. prescribed the following plaster, which was applied over the whole of the affected side of the face:

℞. Emplast. Resinæ flavæ, ℥i.

Resinæ flavæ, ℥ss.

Terebinthinæ venetæ, ℥iij. Liquat. adm.

Tart. Antimonii, ℥jss. ut fiat Emplast.

When this had remained on the face twenty-four hours, the patient experienced an itching, burning sensation throughout the spot covered by it, but the face-ache was relieved. At the end of several days the plaster was taken off, when the entire half of the face was found covered with pustules, which gave a good deal of pain, but which were very bearable in comparison to the former pains. The sores gradually healed by the application of simple dressing, and up to this time (three and a quarter years,) there has been no recurrence of the complaint.

*Medicinische Zeitung*, 6 *Jan.* 1836.

---

*On Sulphate of Quinine introduced into the Nostrils in Iralgia.*
By M. BOURJOT ST. HILAIRE.

THE pain attending some diseases of the eyes is very distressing. In acute inflammatory diseases, opiate applications, such as poppy decoction, or a poultice sprinkled with laudanum, are more serviceable than belladonna or other acrid narcotics. But when, in chronic iritis, there are periodical returns of pain, quinine is of great service. In the following case it was applied within the nostril.

CASE. M. B., æt. sixty, was operated on for cataract in June. The operation, which was by depression, was followed by acute inflammation of the globe, attended with great suffering: this, however, abated, and the patient saw well in August, when, on exposure to the air, he had an attack of iritis, with neuralgia of the sub-orbitary nerve. He was bled in the foot, leeches were applied in large numbers, and purgatives given, without much relief. The pain in the eye was excessive, lancinating, and returning regularly every two hours; more acute at night, preventing sleep. The usual opiate applications were tried without relief, and, as the pain was periodical, M. B. resolved to try quinine by the endermic method. Six grains were twice applied to the raw surface of a blister placed on the nucha. This produced decided and immediate relief, the patient passing the two nights following the application in almost complete tranquillity; but the blistered surface became covered with a white film, and absorption being hindered, the pains returned. Reflecting on the distribution of the nasal branch of the ophthalmic nerve on the mucous membrane of the nostrils, M. B. ordered small pinches of the following powder to be snuffed up the nostrils at night:

℞. Sulph. Quininæ, gr. vj.

Pulv. Sacchar. pur. ʒj.

Pulv. R. Iridis, ʒjss. M. fiat pulvis.

This produced the desired effect, quieting the pain whenever he used it.

*Gazette Médicale de Paris*, 26 *Mars*, 1836. No. 15.

---

*Cure for Drunkenness.*

A NATIVE of Norway, aged forty, who had from his youth been accustomed to dram-drinking, was attacked with delirium tremens. His medical attendant, to cure him of his dangerous propensity, prescribed the daily use of a mixture of two drachms of sulphuric acid and twenty-four ounces of whiskey. The result was remarkable: in three months' time he got such a dislike to all kinds of spirituous liquors, that he could not bear to swallow a drop of any thing stronger than beer. The dose of the above mixture taken was four wine-glasses daily, and the cure had been of a year's standing at the time of the communication of the case.

*Eyr.* Tiende Bind. andet Hefte.

---

*Stuttering occasioned by Worms.* By Dr. SCHULTZE.

A BOY, aged five years, who hitherto could distinctly pronounce even the most difficult words, and readily communicate his thoughts, all at once began to stutter. As no organic defect could be perceived, Dr. S. thought that the impediment might be occasioned by worms, as he had often noticed an entire loss of speech, lasting many days, to depend upon this cause. He therefore ordered an electuary composed of jalap, semin. cinæ, tanacet., and magnes. sulph., with syr. mannæ, to be given. By this medicine, a large quantity of the ascarides lumbricoides were voided, and the boy was again restored to the free use of his speech.

*Med. Zeitung*, 6 *Januar*, 1836.

